# Analyzing Cardiovascular Characteristics of Patients Initially Diagnosed with Breast Cancer in Saudi Arabia

**DOI:** 10.7759/cureus.45799

**Published:** 2023-09-22

**Authors:** Sahar M Alnefaie, Mohammed A Alosaimi, Meshal F Althobaiti, Abdulmajeed A Altowairqi, Mohammed K Alrawqi, Sami M Alzahrani, Ghaliah O Alnefaie, Maryam S Aljaid

**Affiliations:** 1 Department of Surgery, College of Medicine, Taif University, Taif, SAU; 2 College of Medicine, Taif University, Taif, SAU; 3 Department of Surgery, Alhada Armed Forces Hospital, Taif, SAU; 4 Pathology, Taif University, Taif, SAU; 5 Department of Pediatrics, College of Medicine, Taif University, Taif, SAU

**Keywords:** cardiovascular characteristics, hypertension, hyperlipidemia, metastasis, breast cancer

## Abstract

Introduction

Cancer is a condition where abnormal cells proliferate uncontrollably, leading to metastasis, which can be related to death. Breast cancer is the most prevalent type among women worldwide. Early detection with screening mammography has contributed to the decline in breast cancer incidence and mortality. Breast cancer patients are more likely to develop cardiovascular disease, with elderly patients dying from complications. Understanding the patients’ cardiovascular status prior to treatment is essential. The study's objective was to evaluate the cardiovascular characteristics of women with breast cancer at diagnosis within the designated time frame of one year.

Methodology

This was a retrospective study that focused on patients in Taif City, Saudi Arabia, who were initially diagnosed with primary breast cancer over a span of one year. The inclusion criteria encompassed eligible patients, while those not meeting the criteria were excluded. Data extraction from patients’ records was conducted, and the analysis was executed using IBM SPSS Statistics for Windows, Version 26.0 (Released 2019; IBM Corp., Armonk, New York, United States).

Results

This study analyzed the cardiovascular attributes of breast cancer patients, focusing on 136 female cases. The study found significant patterns concerning cardiovascular risk factors in breast cancer patients, categorized by menopausal status. Premenopausal female cases had a mean age of 43.09 ± 8.31 years, while postmenopausal patients had an average age of 58.07 ± 11.70 years. Postmenopausal patients had a higher prevalence of overweight/obesity, irregular menstrual cycles, type II diabetes, hypertension, and hyperlipidemia compared to their premenopausal counterparts. No significant differences were found between the two groups regarding low-density lipoprotein (LDL) cholesterol levels, axillary lymph node metastasis, or distant metastasis. This study emphasized the importance of regular check-ups for menopausal women to detect potential health complications early.

Conclusion

In summary, breast cancer is a global health concern, and understanding its impact on the cardiovascular system is crucial for comprehensive patient care. A study in Saudi Arabia found associations between cardiovascular risk factors and menopausal status in breast cancer patients. Postmenopausal patients had more prevalent risk factors, emphasizing the need for proactive assessment and management. Age-appropriate screenings and interventions are essential. Integrated healthcare approaches should consider the interplay between breast cancer and cardiovascular health, with medical professionals vigilant in evaluating and addressing risk factors to mitigate complications and optimize long-term outcomes.

## Introduction

Cancer is a condition in which a group of abnormal cells proliferate without control by breaking the rules of normal cell division. Signals constantly influence whether normal cells divide, differentiate into another type of cell, or die. Cancer cells acquire some degree of independence from these signals; hence, they replicate in an unregulated manner. It could be fatal if this proliferation is allowed to continue and spread. In fact, metastasis, the spread of a tumor to a distant site, accounts for nearly 90% of cancer-related deaths [1]. Women are more likely to develop breast cancer than men, who account for less than 1% of all cases [2]. Breast cancer in men is uncommon; hence, due to its low incidence, literature, research, clinical trials, and the development of new treatment options primarily focus on female breast cancer. Although knowledge of female breast cancer can help men diagnose and treat breast cancer, male and female breast cancer have distinct molecular and clinicopathologic characteristics. When defining this disease in men and selecting a treatment option, biological factors such as sex differences, hormonal regulation, and response to treatment (both tolerability and activity) must be taken into consideration [3].

Worldwide, breast cancer is the most prevalent malignancy among women. Over two million new cases of breast cancer, or 11.6% of all cancers, were diagnosed worldwide in 2018. Additionally, breast cancer is the leading cause of cancer-related deaths in women [4]. The appropriate application of systemic adjuvant therapy and early detection with screening mammography are two of the many factors that have contributed to the decline in breast cancer incidence and mortality in many developed nations.

Breast self-examination (BSE), clinical breast examination (CBE), and mammography can all be used to detect breast cancer early. Because breast cancer is frequently diagnosed in its later stages, Arabic women currently face a significant risk of a high mortality rate. The prevalence of breast cancer is rising and affecting a younger population in the Middle East and Gulf region compared to the West. There are very few breast cancer education programs in the Arab world [5]. The incidence of breast cancer in the Kingdom of Saudi Arabia (KSA) has been rising in recent years, from 1152 cases per 100,000 people in 2008 to 1473 cases per 100,000 people in 2010 and 1826 cases per 100,000 people in 2014. According to the KSA Health Council’s 2014 cancer registry, breast cancer is the most common cancer in women, accounting for 28.7% of all cancers. Another study found that 13.08% of all deaths were caused by breast cancer, with women accounting for 98% of deaths and men for 12% [6-8].

It has been reported that breast cancer patients are significantly more likely to develop cardiovascular disease [9]. The mortality rate from cardiovascular and cerebrovascular disease complications in elderly breast cancer patients is 15.9%, which is higher than the 15.1% mortality rate from breast cancer recurrence [10]. Cardiovascular disease (CVD) is now the leading cause of death among breast cancer patients [11]. Anthracycline increases the risk of drug-related cardiovascular problems in breast cancer patients with cardiovascular risk factors (CVRFs) [12]. Also, patients with breast cancer had significantly higher rates of CVD and mortality from radiotherapy and chemotherapy [13]. Due to the rising incidence of breast cancer and improved long-term survival rates, cardiovascular disease has emerged as the leading cause of death among breast cancer patients. Breast cancer and CVD share many risk factors and interact with one another [14]. Finally, understanding the cardiovascular status prior to treatment is essential. The majority of studies on CVRFs of breast cancer patients, both domestically and internationally, currently face numerous issues, including a lack of clinical data and incomplete data collection. As a result, it is challenging to conduct a thorough analysis of the CVRFs of breast cancer patients. The CVRFs and arteriosclerotic cardiovascular disease (ASCVD) risk assessment of breast cancer patients in Taif City were the focus of this study.

A few studies have been conducted internationally regarding the cardiovascular characteristics of breast cancer patients at diagnosis, and these studies indicate there is a correlation between CVRFs and axillary lymph node metastasis (ALNM) [14].

## Materials and methods

Study design

This study was a retrospective study conducted between April 2023 and July 2023 in Taif City, Kingdom of Saudi Arabia.

Study population and sampling methodology

This study had a sample size of 136 patients, and the data were gathered from patient files at Alhada Armed Forces Hospital in Taif City, Saudi Arabia. The inclusion criteria were all consecutive patients diagnosed with initial primary breast cancer from January 2017 to January 2022. In addition, patients with missing clinical data within their medical files or those diagnosed outside the period under consideration were excluded. Data were collected through a previously validated study by Dong Z in 2021 [14].

Data analysis

The analysis was performed using the latest version of IBM SPSS Statistics for Windows, Version 26.0 (Released 2019; IBM Corp., Armonk, New York, United States). Descriptive statistics such as mean, standard deviation, frequency, and percentage were used to summarize independent variables. Qualitative data was transformed into percentage mean scores. Quantitative data was analyzed using the chi-square test and analysis of variance (ANOVA), while qualitative data was analyzed using the chi-square test and student t-test. A p-value < 0.05 indicated statistical significance.

Ethical considerations

Ethical approval was provided by the Institutional Review Board (IRB) of Alhada Armed Forces Hospital, Taif (REC.2023-722). The study was performed in accordance with the Declaration of Helsinki, and written informed consent was waived due to its retrospective nature.

## Results

In this retrospective cross-sectional study, a mass screening involving 136 individuals was conducted in Taif city, Saudi Arabia, over a period of a year. The primary aim was to evaluate the cardiovascular attributes of individuals who were already diagnosed with breast cancer. A thorough analysis of descriptive data was done to assess the prevalence rates of cardiovascular risk factors in individuals previously diagnosed with breast cancer. Subsequently, the study investigated significant correlations among various variables utilizing both t-test and Chi-square test methodologies, employing a significance threshold of 0.05. Finally, a binary multivariate logistic regression analysis was executed to find out potential risk factors associated with axillary lymph node metastasis among patients initially diagnosed with breast cancer.

Characteristics of the study population

**Figure 1:**
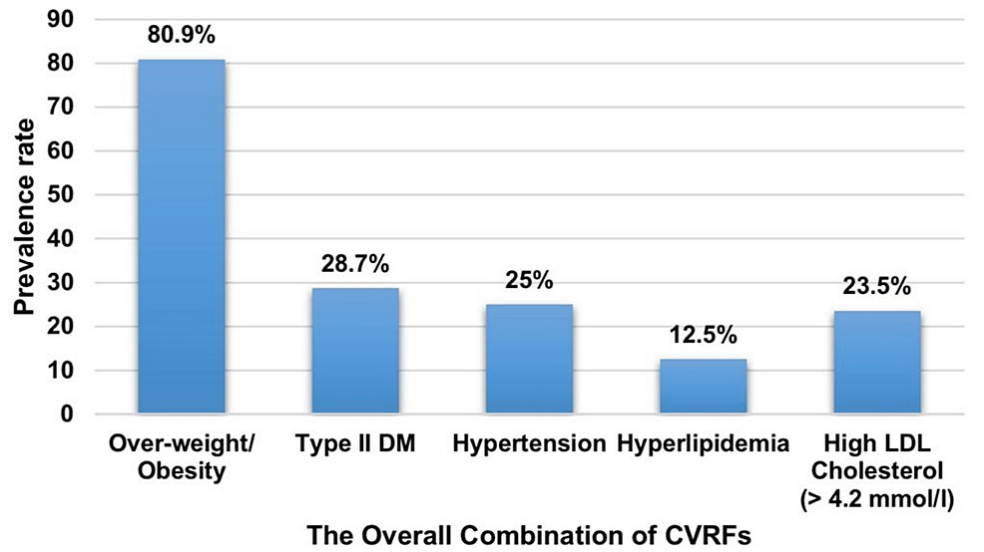
Prevalence rates of cardiovascular risk factors in breast cancer cases (n = 136) CVRFs: cardiovascular risk factors

As depicted in Figure 1, among the cohort of 136 female patients diagnosed with primary breast cancer, the average age stood at 50.58 ± 12.60 years, ranging from 25 years to 110 years. Of these patients, exactly half (n = 68) were categorized as premenopausal. In terms of body weight, a significant proportion of the participants (n = 110, 80.9%) were identified as either overweight or obese. Additionally, 73 patients (53.7%) had irregularities in their menstrual cycles, while 39 (28.7%) were diagnosed with type 2 diabetes mellitus. In addition, 34 patients (25%) presented with hypertension, 17 patients (12.5%) had hyperlipidemia, and 32 patients (23.5%) exhibited elevated low-density lipoprotein (LDL) cholesterol levels exceeding 4.2 mmol/L.

Combination of cardiovascular risk factors (CVRFs) in premenopausal and postmenopausal patients with breast cancer 

The data presented in Table 1 reveals significant patterns concerning the combination of cardiovascular risk factors in patients with breast cancer, categorized by their menopausal status. Premenopausal female patients exhibited a mean age of 43.09 ± 8.31 years, while their postmenopausal counterparts had an average age of 58.07 ± 11.70 years. Notably, statistically significant differences were observed between the two groups. Postmenopausal patients displayed a considerable increase in the prevalence of overweight/obesity (p value = 0.006), irregular menstrual cycle (p value = 0.001), type 2 diabetes mellitus (p value = 0.001), hypertension (p value = 0.001), and hyperlipidemia (p value = 0.004), compared to their premenopausal counterparts. However, no statistically significant differences were found between the two groups regarding LDL cholesterol levels, axillary lymph node metastasis, or distant metastasis (p > 0.05).

**Table 1:** Characteristics of cases with initial breast cancer (n = 136) LDL: low-density lipoprotein; ALNM: axillary lymph node metastasis

Variable	All n (%)	Premenopausal group (n = 68)	Postmenopausal group (n = 68)	t/χ^2^	p-value
Age	50.58 ±12.60	43.09 ±8.31	58.07 ±11.70	-8.605	0.001
Over-weight/ obesity	110 (80.9)	49 (72.06)	61 (89.7)	14.584	0.006
Irregular menstrual cycle	73 (53.7)	26 (38.24)	47 (69.11)	13.041	0.001
Type 2 diabetes mellitus	39 (28.7)	10 (14.70)	29 (42.64)	12.978	0.001
Hypertension	34 (25.0)	8 (11.76)	26 (38.23)	12.706	0.001
Hyperlipidemia	17 (12.5)	3 (4.41)	14 (20.58)	8.134	0.004
High LDL cholesterol (> 4.2 mmol/l)	32 (23.5)	15 (22.06)	17 (25.0)	1.101	0.577
Axillary lymph node metastasis (ALNM)	105 (77.2)	49 (72.06)	56 (82.35)	2.047	0.152
Distant metastasis	27 (19.9)	10 (14.70)	17 (25.0)	2.264	0.132

Similarly, as depicted in Table 2, female patients with breast cancer in the Taif region had a relatively younger age at diagnosis (47.91 ± 10.43) compared to those residing in other cities (51.90 ± 13.40). Additionally, a substantial increase in the number of cases among postmenopausal patients was observed outside the Taif region (p value = 0.002). Furthermore, the prevalence of overweight and/or obese patients was notably higher in areas outside Taif (p value = 0.044). Conversely, no statistically significant variations were evident in other risk factors that could impact breast cancer cases, whether inside or outside the Taif region (p > 0.05).

**Table 2:** Characteristics of patients with breast cancer inside and outside Al-Taif (n = 136) BC: breast cancer; LDL: low-density lipoprotein; ALNM: axillary lymph node metastasis

Variable	BC cases in Taif (n = 45)	BC cases outside Taif (n = 91)	t/χ^2^	p-value
Age	47.91 ±10.43	51.90 ±13.40	-1.750	0.082
Postmenopausal state	14 (31.11)	54 (59.34)	9.598	0.002
Over-weight/ obesity	33 (72.0)	77 (89.7)	9.804	0.044
Irregular menstrual cycle	23 (35.6)	50 (64.4)	0.178	0.673
Type 2 diabetes mellitus	12 (25.6)	27 (74.4)	0.133	0.716
Hypertension	11 (23.5)	23 (76.5)	0.011	0.916
Hyperlipidemia	4 (17.6)	13 (82.4)	0.802	0.371
High LDL cholesterol (> 4.2 mmol/l)	12 (46.9)	20 (53.1)	1.238	0.539
Axillary lymph node metastasis (ALNM)	36 (46.7)	69 (53.3)	0.298	0.585
Distant metastasis	11 (37.0)	16 (63.0)	0.891	0.345

Correlation analysis of cardiovascular risk factors (CVRFs) and axillary lymph node metastasis (ALNM) in breast cancer patients

The relationship between cardiovascular risk factors (CVRFs) and the occurrence of axillary lymph node metastasis (ALNM) in patients with breast cancer was investigated through correlation analysis, as outlined in Table 3. The results of the binary logistic regression analysis, detailed in the table, indicated that none of the examined cardiovascular risk factors exhibited a statistically significant impact on the occurrence of axillary lymph node metastasis (p > 0.05).

**Table 3:** Binary logistic regression analysis of factors related to axillary lymph node metastasis (ALNM) in cases with initial breast cancer (n = 136) OR: odds ratio, CI: confidence interval; BMI: body mass index; LDL: low-density lipoprotein; ALNM: axillary lymph node metastasis

Variable	OR	95% CI	p-value
Age	1.018	0.587-1.766	0.948
Body mass index (BMI)	1.380	0.878-2.167	0.163
Residence area	0.720	0.270-1.918	0.511
Number of children	1.538	0.783-3.021	0.212
Menstrual cycle	1.097	0.695-1.730	0.691
Menopause	1.138	0.613-2.110	0.682
Type 2 diabetes mellitus	1.880	0.809-4.368	0.142
Hypertension	1.013	0.507-2.021	0.971
Hyperlipidemia	0.543	0.193-1.525	0.246
LDL cholesterol level	1.159	0.656-2.049	0.611
Distant metastasis	1.942	0.887-4.251	0.097

To sum up, in contrast to their premenopausal counterparts, postmenopausal patients diagnosed with breast cancer tend to display an increased prevalence of cardiovascular risk factors and a higher susceptibility to arteriosclerotic vascular diseases. Moreover, no significant relationships have been established between cardiovascular risk factors and the occurrence of axillary lymph node metastasis in breast cancer patients. These findings underscore the importance of regular check-ups for menopausal women, enabling the timely detection of potential health complications.

## Discussion

Our study delves into the cardiovascular characteristics of patients initially diagnosed with breast cancer in Saudi Arabia, shedding light on important associations between menopausal status, cardiovascular risk factors, and axillary lymph node metastasis.

The demographic characteristics of the study population reveal a notable prevalence of overweight and obesity, irregular menstrual cycles, type 2 diabetes mellitus, and hypertension among breast cancer patients. These findings underscore the importance of assessing cardiovascular risk factors in breast cancer patients, as these factors could potentially influence disease outcomes and treatment strategies. The prevalence of these risk factors in this study aligns with those of previous studies that have reported similar patterns of cardiovascular risk factors in breast cancer patients, indicating the consistency of these associations across different populations [15].

The study’s exploration of the combination of cardiovascular risk factors in premenopausal and postmenopausal breast cancer patients provides a nuanced perspective. Premenopausal patients displayed a notably younger mean age compared to their postmenopausal counterparts, underscoring the expected correlation between menopause and advancing age. This aligns with the findings of prior studies, which reported similar age discrepancies between menopausal stages in breast cancer patients [16]. The statistically significant disparities in the prevalence of cardiovascular risk factors between the two groups, including overweight/obesity, irregular menstrual cycles, type II diabetes mellitus, hypertension, and hyperlipidemia, further substantiate the impact of menopausal status on these risk factors. These findings are consistent with those of the research, which highlighted the pronounced influence of the menopausal transition on cardiovascular health parameters [17]. Interestingly, the absence of statistically significant differences in LDL cholesterol levels, axillary lymph node metastasis, or distant metastasis between the two groups suggests that while menopause appears to correlate with increased cardiovascular risk factors, it might not inherently affect these specific breast cancer-related aspects. This notion is supported by a study that found no direct connection between menopausal status and axillary lymph node metastasis [18]. These findings collectively contribute to a more comprehensive understanding of the intricate relationship between menopausal status, cardiovascular risk factors, and breast cancer outcomes.

Studies from the cities outside the Taif region provide insights into potential regional variations. Notably, breast cancer patients in the Taif region were diagnosed at a relatively younger age compared to patients from other cities. This discrepancy echoes the findings of a study that highlighted age-related disparities in breast cancer diagnosis across different regions [19]. The observed substantial increase in postmenopausal cases outside the Taif region aligns with the notion that regional variations may impact the prevalence of menopausal stages among breast cancer patients. This is in line with the findings of the research, which emphasized the role of geography in menopausal transition and its implications for breast cancer cases [20].

Furthermore, the significantly higher prevalence of overweight and/or obese patients outside the Taif region points to potential disparities in lifestyle and healthcare access. This result is consistent with that of studies that emphasized the role of geographic factors in shaping health behaviors and outcomes [21]. Interestingly, the absence of statistically significant variations in other risk factors impacting breast cancer cases, both within and outside the Taif region, highlights the nuanced nature of these regional differences. These findings underscore the significance of geographic-specific interventions aimed at addressing obesity rates and potentially reducing the associated breast cancer risk. Additionally, they underscore the importance of considering multiple factors, such as healthcare access, lifestyle, and cultural influences, in understanding regional disparities in breast cancer characteristics and outcomes [22,23].

The investigation into the correlation between cardiovascular risk factors and axillary lymph node metastasis adds depth to the understanding of disease progression. The lack of statistically significant relationships between these risk factors and axillary lymph node metastasis suggests that while cardiovascular risk factors may influence overall health, they may not directly impact the metastatic spread of breast cancer [24]. This insight highlights the multifaceted and multifactorial nature of metastasis, suggesting that its mechanisms extend beyond the scope of cardiovascular risk factors [25]. The complexity of this phenomenon necessitates ongoing research endeavors aimed at unraveling the underlying intricacies governing metastasis. By delving deeper into these mechanisms, researchers can uncover novel insights that could potentially pave the way for more targeted therapeutic interventions focused on mitigating the spread of breast cancer.

Study limitations

Despite the valuable insights provided by this research, it is important to acknowledge certain limitations that may impact the interpretation and generalizability of the findings. Firstly, the study’s sample size of 136 individuals from a single city, Taif, might not fully represent the diverse population of breast cancer patients across Saudi Arabia; the relatively small sample could limit the generalizability of the results to a broader context.

## Conclusions

In summary, understanding the effects that breast cancer has on the cardiovascular system is essential for providing tailored patient treatment. Breast cancer is a problem that affects the health of people all over the world. Researchers in Taif, Saudi Arabia, looked at breast cancer patients and observed connections between breast cancer patients' menopausal status and cardiovascular risk variables. Patients who had gone through postmenopause had a higher prevalence of cardiovascular risk factors, which highlighted the importance of early examination and therapy. It is vital to conduct tests and therapies that are suitable for the patient's age. Integrated healthcare approaches should take into account the interaction between breast cancer and cardiovascular health, and medical professionals should be alert when evaluating and resolving risk factors in order to reduce the likelihood of complications and maximize the likelihood of positive long-term results. This research makes a significant contribution to the expanding body of knowledge concerning breast cancer's broader influence on patient health. As a result, medical professionals now have the ability to modify strategies for the early detection, prevention, and management of breast cancer and associated cardiovascular risks. It is crucial to take a holistic approach to patient care, placing a priority on the patient's overall health and well-being when treating breast cancer.
